# Ribosome-engineered *Lacticaseibacillus rhamnosus* GG with enhanced adhesion and immune activation via surface moonlighting proteins

**DOI:** 10.1128/spectrum.02635-25

**Published:** 2025-10-27

**Authors:** Masami Tsukagoshi, Jamiyanpurev Soyolmaa, Shunsaku Nomoto, Fu Namai, Aito Murakami, Kazuma Inoue, Masahiro Yoda, Tomoyuki Hibi, Takashi Sato, Hideki Kinoshita, Takeshi Shimosato

**Affiliations:** 1 Department of Agriculture, Graduate School of Science and Technology, Shinshu University 13056, Minamiminowa, Kamiina, Nagano, Japan; 2 Food and Feed Immunology Group, Laboratory of Animal Food Function, Graduate School of Agricultural Science, Tohoku University 13101, Sendai, Miyagi, Japan; 3 Graduate School of Medicine, Science and Technology, Shinshu University 13056, Minamiminowa, Kamiina, Nagano, Japan; 4 Institute for Biomedical Sciences, Shinshu University, Minamiminowa, Kamiina, Nagano, Japan; 5 Institute for Aqua Regeneration, Shinshu University 13056, Minamiminowa, Kamiina, Nagano, Japan; 6 Faculty of Agriculture, Shinshu University, Minamiminowa, Kamiina, Nagano, Japan; 7 Graduate School of Bioscience, Tokai University 497074, Mashiki-machi, Kumamoto, Japan; University of Minnesota Twin Cities, St. Paul, Minnesota, USA

**Keywords:** *Lacticaseibacillus rhamnosus* GG, probiotics, ribosome engineering, *rpsL*, cell surface protein, GAPDH

## Abstract

Commercial interest in using probiotics to enhance human health and animal production is growing. Accordingly, there is great demand for probiotic lactic acid bacteria exhibiting enhanced robustness and functionality. The present study focused on ribosome engineering (RE), a simple, economical, and safe non-genetically modified organism microbial breeding technology. Ribosome-engineered mutants of *Lacticaseibacillus rhamnosus* GG (LGG), one of the most widely used probiotic strains, were characterized. We found that an LGG strain with the K56N mutation within the *rpsL* gene (MT_K56N_: K56N) expressed a variety of proteins, primarily moonlighting proteins, on the cell surface, likely due to increased translational efficiency and/or activated protein transport via ABC transporters. The K56N mutation is suggested to be a major contributing factor to the enhanced expression of moonlighting proteins, although contributions from additional mutations cannot be ruled out. The K56N surface proteins, including DnaK, GroEL, PyK, and GAPDH, mediated strong bacterial adhesion to intestinal cells and exerted immunostimulatory effects in macrophages by enhancing tumor necrosis factor (TNF)-α production. Interestingly, although LGG–wild-type extracellular vesicles (EVs) suppressed lipopolysaccharide-induced TNF-α production, this effect was not observed with K56N-EV. These findings highlight the potential use of K56N as a probiotic strain with immune-activating properties and the feasibility of using RE to develop probiotic strains for future industrial applications.

**IMPORTANCE:**

Ribosome engineering, a microbial breeding strategy that induces spontaneous mutations via treatment with antibiotics, has led to novel functionalities in *Lacticaseibacillus rhamnosus* GG. A study of streptomycin-resistant *rpsL* mutants identified the K56N mutation as a key driver of enhanced protein display on the bacterial surface. This mutation promoted strong adhesion to intestinal cells and immunostimulatory effects in macrophages. The mutation also triggered transcriptomic shifts and morphologic changes, highlighting the broad range of bacterial modifications.

## INTRODUCTION

There is growing commercial interest in the use of probiotics (defined as “live microorganisms that, when administered in adequate amounts, confer a health benefit on the host”) (1). Lactic acid bacteria (LAB) are industrially important microorganisms with a long history of safe use; a number of LAB strains have been recognized as probiotics that inhibit pathogenic bacteria, restore gut microbiota, improve epithelial barrier properties, and modulate immune responses (2). However, even bacterial strains exhibiting industrially promising properties can be unsuitable for practical use without the introduction of specific improvements (3). Probiotic efficacy can be affected by a range of factors, including host genetic/environmental background, structure of the intestinal microbiota, and administration dose/schedule (4–7). Accordingly, strains that exhibit enhanced robustness and functionality are favored for industrial applications.

Bacterial strains can be improved using a variety of methods, such as random mutagenesis, adaptive evolution, and genetic engineering. In 2020, Rasinkangas et al. applied chemical mutagenesis to one of the most studied probiotic strains, *Lacticaseibacillus rhamnosus* GG (LGG), to generate derivatives exhibiting enhanced adherence to mucin (8). However, the application of such classical methods can be hampered by various challenges, including safety risks to handlers (9), the occurrence of unintended or undesired mutations (3), and consumer concerns regarding genetically modified organisms (GMOs) (10, 11). Ribosome engineering (RE) represents a promising alternative strategy characterized by the introduction of spontaneous mutations using antibiotics (12). RE specifically targets RNA polymerase or ribosomes to alter transcription or translation (13), enhances secondary metabolite production, and was shown to enable the production of novel metabolites in *Streptomyces*, *Bacillus*, and *Escherichia* (14–17). RE is a simple, economical, and safe non-GMO breeding technology for microorganisms, which does not require any specialized facilities or equipment.

The bacterial proteosurfaceome, defined as “the proteinaceous subset of the surfaceome found at the cell wall and totally or partially exposed on the external side of the cell membrane” (18), has received increased research interest to enhance understanding of host-probiotic interactions (19). Notably, intracellular anchorless proteins, such as metabolic enzymes and molecular chaperones, reportedly impart additional functions on the bacterial surface (18, 19). Such proteins, also referred to as moonlighting proteins, include glyceraldehyde-3-phosphate dehydrogenase (GAPDH) and enolase, which moonlight as an adhesin and an immunomodulatory molecule on the cell surface of *Lactobacillaceae* (20, 21). In recent years, bacterial surface molecules have even been recognized as potential targets for technological and functional improvements of probiotics (22).

In our previous study, we demonstrated the utility of RE as a novel breeding strategy for probiotic LAB by applying it to LGG. We isolated eight streptomycin-resistant mutants (MTs) harboring distinct point mutations in the *rpsL* gene, which encodes ribosomal protein S12. Among them, the K56N mutant displayed increased expression of the moonlighting protein GAPDH on its surface and showed enhanced adhesion to human colonic mucin via GAPDH-mediated binding (23). While our prior work focused primarily on the adhesion phenotype, the broader functional consequences of RE-induced alterations in surface protein profiles remain unclear. The present study extends this investigation by exploring how the K56N mutation affects extracellular vesicle (EV) composition and host immune modulation. Through detailed proteomic and immunological analyses, we uncover a novel role for EV-associated GAPDH as an immunostimulatory factor, providing new mechanistic insights into host-microbe interactions mediated by RE-derived probiotic strains.

## MATERIALS AND METHODS

### Ribosome engineering of LGG

RE was applied to LGG as previously described (23). In our earlier study, we reported eight ribosome-engineered LGG mutants (23). In the present work, we additionally generated a novel mutant (R99G) through repeated selection, which was not included in the previous report. Briefly, LGG–wild-type (WT) was grown in de Man, Rogosa, and Sharpe (MRS) broth at 37°C overnight. The resulting culture (1 × 10^8^ colony-forming units [CFU]/500 µL) was grown at 37°C for 3 days on MRS agar containing streptomycin at the minimum inhibitory concentration (MIC; 256 µg/mL). All individual colonies were assigned an identification number and cultured at 37°C for 3 days on MRS agar containing 0 µg/mL streptomycin. Isolates were subsequently cultured at 37°C for 3 days on MRS agar containing 256 µg/mL streptomycin to obtain streptomycin-resistant LGG MTs. The *rpsL* gene was amplified from each isolate and subjected to DNA sequencing using the sense primer 5′- GGC TGA CGC ATA TTC TGT CTA TAC CG -3′ and anti-sense primer 5′- GTT GTC CGG ACG TGC TGA CT -3′. Growth of the WT and MT strains was monitored by optical density (OD) measurement at a wavelength of 660 nm (OD_660_) every hour for 24 h using a spectrophotometer. OD_660_ values were determined for cultures grown in MRS broth without streptomycin.

### Bacteria and cultures

LGG (ATCC 53103; ATCC, VA, USA) WT parent (LGG-WT/WT) and ribosome-engineered mutants (LGG-MTs/MTs: K56N, K101E, K56T, K101R, K56M, K56R, K101M, and R99G obtained in our previous study [23], and R99C obtained in this study) were used (Table 1). WT and MT strains were pre-cultured statically at 37°C for 16 h in MRS broth.

**TABLE 1:** Strains used in this study^*a*^

Colony no.	Relevant genotype	Frequency of mutants	MIC (µg/mL)
WT	−^*b*^	−	256
#001	*rpsL* (K56N)	58/136	>65,536
#006	*rpsL* (K101E)	41/136	4,096
#010	*rpsL* (K56T)	8/136	>65,536
#022	*rpsL* (K101R)	4/136	2,048
#027	*rpsL* (K56M)	5/136	>65,536
#045	*rpsL* (K56R)	14/136	>65,536
#112	*rpsL* (K101M)	4/136	4,096
#134	*rpsL* (R99C)	1/136	512
#157	*rpsL* (R99G)	1/136	2,048

^*a*^ Frequency of mutants: number of events among the 136 confirmed LGG-MTs that were isolated.

^*b*^ −, no mutations detected in the wild-type strain.

### Morphological observations using scanning electron microscopy (SEM)

Pre-cultures of WT and MT strains were centrifuged (8,000 × *g*, 4°C, 5 min) to harvest bacterial cells. The cell pellets were washed (8,000 × *g*, 4°C, 5 min) with phosphate-buffered saline (PBS) at pH 7.3 (PBS_pH7.3_) and resuspended in the same. The resulting suspensions were placed onto Nanopercolator filters (JEOL, Tokyo, Japan), fixed in 2.5% glutaraldehyde (TAAB Laboratories Equipment, Ltd., Berks, UK) for 1 h, washed three times with 0.1 M cacodylate buffer, and immersed in a mixture of 0.1 M cacodylate buffer and 1% osmium (NEM, Tokyo, Japan) for 1 h. Following a wash with sterile water, the plasma membranes were dehydrated using an ethanol series (50%, 60%, 70%, 80%, 90%, 95%) for 5 min each, followed by three times in 100% ethanol for 30 min each. After two rounds of immersion in t-butyl alcohol (Nacalai Tesque) at 52°C for 30 min, the membranes were stored at −80°C. The samples were then lyophilized using a DC401 freeze dryer (Yamato Scientific, Tokyo, Japan), coated with osmium using a Neoc-AN (Meiwafosis Co., Ltd., Tokyo, Japan), and observed using a JSM-7600F scanning electron microscope (JEOL).

### Preparation of bacterial surface protein extracts

Pre-cultures of WT and MT strains were inoculated into MRS broth at OD_600_ = 0.02 and grown at 37°C. Aliquots (5 mL) of the cultures were centrifuged, and the supernatants were removed to obtain cells. The cell pellets were washed with 1 mL of PBS_pH4.2_ by centrifugation (8,000 × *g*, 4°C, 5 min) and resuspended in 1 mL of either PBS_pH7.3_ or PBS_pH4.2_ and centrifuged (20,600 × *g*, 4°C, 15 min) to collect the supernatants as bacterial lavage fluid (BLF)_pH7.3_ or BLF_pH4.2_. Surface-associated proteins were extracted essentially as described in our previous study (23), which was adapted from established protocols for surface protein extraction in lactic acid bacteria (20).

### Analysis of surface protein profiles

BLF samples (10 mL culture equivalents collected at 6, 9, 12, and 24 h) were clarified to minimize contamination with intracellular proteins. Proteins were precipitated with trichloroacetic acid, washed with acetone, resuspended in NaOH, and analyzed by SDS-PAGE and Coomassie brilliant blue staining. BLF was prepared from 24 h cultures standardized to 1 × 10^9^ CFU/mL. Equal loading for SDS-PAGE and Western blot analyses was ensured by preparing BLF fractions from equivalent CFU-normalized cell suspensions, so that comparable protein amounts were analyzed across samples. The total protein concentration was determined using a Pierce bicinchoninic acid (BCA) Protein Assay Kit (Thermo Fisher Scientific, MA, USA). BLF from 24 h cultures (10 mL) was dialyzed, lyophilized, and subjected to liquid chromatography–mass spectrometry (LC-MS) analysis using liquid chromatography–mass spectrometry with an ion trap–time-of-flight analyzer (LCMS-IT-TOF) and matrix-assisted laser desorption/ionization–time-of-flight mass spectrometry (MALDI-TOF MS) (Tokai University). Proteomic analyses were performed in three independent biological replicates. Mascot ion scores were calculated as –10 × log(*P*), where *P* is the probability that the observed match is a random event. Individual ion scores greater than 24 indicate identity or extensive homology (*P* < 0.05). Protein scores were derived from ion scores as a non-probabilistic ranking of protein hits. Only proteins with Mascot scores above this threshold and consistently detected in at least two of the three biological replicates were considered high-confidence identifications and used for downstream analyses.

### Western blotting of surface GAPDH and elongation factor thermos unstable (EF-Tu)

Protein solutions prepared from BLF_pH7.3_ were subjected to SDS-PAGE using 15% (vol/vol) polyacrylamide gels. Proteins were then transferred onto polyvinylidene difluoride (PVDF) membranes (GE Healthcare, Buckinghamshire, Japan) and blocked with skim milk. The membranes were reacted with primary antibody (polyclonal rabbit anti-GAPDH [1/5,000; GeneTex, CA, USA] or monoclonal mouse anti-EF-Tu [1/5,000; Hycult Biotech, Uden, Netherlands]), followed by incubation with secondary antibody (anti-rabbit IgG [1/5,000; Sigma-Aldrich, St. Louis, MO, USA] or anti-mouse IgG [1/5,000; BioLegend, CA, San Diego, CA, USA]). The immunoblots were visualized using ECL Prime Western Blotting Detection Reagent (GE Healthcare) and an ImageQuant LAS500 system (GE Healthcare).

### Transcriptome analysis by RNAseq

Pre-cultures of WT and MT strains were inoculated into MRS broth at a dilution of 1/20 (vol/vol) and grown at 37°C for 24 h. The bacterial cells were harvested by centrifugation (8,000 × *g*, 4°C, 5 min) and then treated with lysozyme (Nacalai Tesque) at 37°C for 10 min at a final working concentration of 2 mg/mL. Total RNA was extracted using a NucleoSpin RNA Kit (Macherey-Nagel) according to the manufacturer’s instructions. The quality of each RNA sample was assessed using a Qubit RNA IQ Assay Kit (Thermo Fisher Scientific) and Qubit 4 fluorometer (Thermo Fisher Scientific). Ribosomal RNA was removed using a RiboMinus Transcriptome Isolation Kit, Bacteria (Thermo Fisher Scientific), and a cDNA transcriptome library was constructed using an Ion Total RNA-Seq Kit, v.2 (Thermo Fisher Scientific). The quality of each cDNA was examined using Agilent DNA 1000 reagents (Agilent Technologies, Inc., Waldbronn, Germany) and an Agilent 2100 Bioanalyzer. RNA was sequenced on an Ion Chef Instrument and Ion GeneStudio S5 system. Data were analyzed using Galaxy software, version 21.09.1.dev0 (https://usegalaxy.org/). Raw read count and normalized gene count (fragments per kilobase of transcript per million mapped reads) were calculated using ht-seq and Cufflinks, respectively. Principal component analysis (PCA) was carried out using RStudio (Boston, MA, USA). Differentially expressed genes (DEGs) were identified at log2 |fold change| > 1 and then subjected to Kyoto Encyclopedia of Genes and Genomes (KEGG) pathway analysis using DAVID Bioinformatics Resources 6.8.

### Metabolome analysis

Twenty-four-hour cultures of WT and K56N were poured over the entire surface of membrane filters (Merck Millipore Corp., Burlington, MA, USA) set in filter folders for decompression filtration (Merck Millipore Corp.), ensuring that the medium containing the bacteria covered the filter surface, after which the medium was completely aspirated. The bacteria were then washed by pouring and aspirating 10 mL of MilliQ water. A total of 1,600 µL of methanol was then added to Petri dishes, and the membrane filters were immersed in the methanol with the bacteria-attached side facing down. The Petri dishes were covered and sonicated for 30 s in an ultrasonic cleaner (SND Corp., Nagano, Japan). After confirming that the bacteria were completely detached from the membrane filter, 1,100 µL of internal standard solution for metabolite extraction (Human Metabolome Technologies, Inc.) was added to the Petri dishes, mixed by pipetting, and allowed to stand at room temperature for 30 s. Next, 2,000 µL of each mixture was collected in 15 mL centrifuge tubes (Nippon Genetics) and centrifuged (2,300 × *g*, 4°C, 5 min). After centrifugation, 350 µL at a time of each supernatant was transferred into the filter cups of the ultrafiltration units. The ultrafiltration units were then centrifuged (9,100 × *g*, 4°C, 2 ~ 5 h) until the liquid in the filter cups was exhausted, and the filtrate was collected. The filtrates were then subjected to metabolome analysis performed by Human Metabolome Technologies, Inc.

### Cells and cell culture

HT-29 human colonic epithelial cells (ATCC HTB-38) and RAW 264.7 mouse macrophages (ATCC TIB-71) were used in this study. Both cell lines were cultured in Dulbecco’s Modified Eagle’s Medium (DMEM; Thermo Fisher Scientific) containing 10% fetal bovine serum (GE Healthcare), a mixture of 100 U/mL penicillin and 100 µg/mL streptomycin (Nacalai Tesque), and non-essential amino acids (Nacalai Tesque) (complete DMEM). Cells were cultured at 37°C in a 5% CO_2_ environment and passaged every 3 days upon reaching 80%–90% confluence in the flask.

### Evaluation of adherence to HT-29 cells

Pre-cultures of WT and MT strains were inoculated into MRS broth at a dilution of 1/20 (vol/vol) and grown at 37°C for 24 h. Bacteria were harvested by centrifugation (8,000 × *g*, 4°C, 5 min) and washed twice with either PBS_pH4.2_ or PBS_pH7.3_. The cell pellets were resuspended in DMEM to obtain 1 × 10^9^ CFU/mL of WT, MT-Cell_pH4.2_ or WT, MT-Cell_pH7.3_. HT-29 cells were seeded in a 24-well plate at 2 × 10^5^ cells/mL/well and cultured at 37°C in a 5% CO_2_ environment for approximately 3 weeks. The culture medium was replaced every 3 days during the first week and every 2 days after the first week. A total of 1 mL of each WT, MT-Cell suspension was added to each well of HT-29 cells and incubated for 1.5 h. Subsequently, the supernatants were discarded. The wells were gently washed twice with 1 mL of PBS_pH4.2_ to remove any non-adherent bacteria and then treated with 100 µL of trypsin-EDTA (0.25%), phenol red (Thermo Fisher Scientific), at 37°C for 10 min to collect adherent bacteria. Bacteria were counted after serial dilution and plating on MRS agar, followed by incubation at 37°C for 48 h. The adhesion rate was calculated as follows:


bacterialadhesionrate=(CFUofadheredbacteria/CFUofinitialbacteriaadded)

### Proteinase K (ProK) treatment of BLF_pH7.3_

WT and MT strains were cultured for 24 h, and BLF_pH7.3_ was prepared from 2 mL aliquots of each culture, as described above. Each BLF_pH7.3_ sample was subjected to protein digestion by treatment with ProK (Sigma-Aldrich) at 37°C for 15 min at a final working concentration of 400 µg/mL, followed by incubation with protease inhibitor cocktail (Roche, Mannheim, Germany) on ice for at least 1 h at a final working concentration of 600 µg/mL (referred to as ProK-treated BLF_pH7.3_).

### Fractionation of BLF_pH7.3_

WT and MT strains were cultured for 24 h, and BLF_pH7.3_ was prepared from 2 mL aliquots of cultures, as described above. BLF_pH7.3_ was placed onto an Amicon Ultra-2 Centrifugal Filter Device 50,000 NMWL (Merck Millipore Corp.) and centrifuged (3,000 × *g*, 4°C, 30 min). The resulting suspension was collected, and PBS_pH7.3_ was added up to 2 mL. The same procedure was repeated twice to obtain BLF containing fractionated proteins of molecular weight >50 kDa. Likewise, Amicon Ultra-2 Centrifugal Filter Devices 30,000 NMWL (Merck Millipore Corp.) were used to obtain BLF containing fractionated proteins of molecular weight between 30 kDa and 50 kDa.

### Bacteria, growth conditions, and plasmid for genetically modified LAB (gmLAB) construction

*Lactococcus lactis* subsp. *cremoris* NZ9000 (NZ9000; MoBiTec, Göttingen, Germany) was employed as the host for putative immunomodulatory protein expression. NZ9000 cells were cultured in M17 broth (BD Difco, Becton, Dickinson and Company, Sparks, MD, USA) containing 0.5% glucose (GM17) at 30°C without shaking. Constructed gmLAB were cultured in GM17 containing 10 µg/mL chloramphenicol (GM17cm), as previously described (24). Plasmid pNZ8148#2 was used as a gene expression vector (24).

### Construction of gmLAB and induction of DnaK, GroEL, Pyk, and GAPDH

Putative immunomodulatory protein genes were amplified from LGG and subjected to DNA sequencing using originally designed primers with *Kpn*I, *Bam*HI, and *Hin*dIII restriction enzyme recognition sites. Each gene segment was then excised using *Kpn*I, *Bam*HI, and *Hin*dIII and cloned into the multi-cloning site of pNZ8148#2. The resulting vectors were transformed into competent *Escherichia coli* MC1061 cells, and the plasmids were isolated from MC1061 using a FastGene Plasmid Mini Kit (Nippon Genetics, Tokyo, Japan). The resulting expression vectors (pNZ8148#2SEC-DnaK, pNZ8148#2SEC-GroEL, pNZ8148#2SEC-Pyk, and pNZ8148#2CYT-GAPDH) were sequenced by Eurofins Genomics (Tokyo, Japan) to confirm the absence of mutations and/or deletions of the target genes. Each expression vector was introduced via electroporation into strain NZ9000 to generate the gmLAB (designated NZ-DnaK, NZ-GroEL, NZ-Pyk, NZ-GAPDH), as previously described (25, 26). pNZ8148#2SEC and CYT were also introduced into strain NZ9000 to generate vector control gmLAB (designated NZ-VC). Each gmLAB strain was pre-cultured in GM17cm overnight, inoculated into 2 mL of pre-warmed GM17cm, and incubated until the OD_600_ reached approximately 0.4. The gene expression inducer nisin was added at a final concentration of 1.25 ng/mL, and the cells were re-incubated for another 3 h. After incubation, each culture was centrifuged (8,000 × *g*, 4°C, 5 min) to obtain cells, which were washed with 1 mL of Tris-buffered saline (TBS) and resuspended in 400 µL of TBS containing protease inhibitor. The resulting suspensions were transferred to screw-cap microcentrifuge tubes containing glass beads (0.2 mmφ) and crushed using a beads-beater (μ-12, TAITEC, Saitama, Japan). The resulting soluble fractions were then mixed with equal volumes of sample buffer (2ME+) (×2) and boiled at 95°C for 5 min.

### Western blotting of secreted proteins

Samples were prepared and subjected to SDS-PAGE as described above using 7.5% (vol/vol) polyacrylamide gels. Proteins were then transferred onto PVDF membranes and blocked with 5% skim milk for 1 h. The membranes were reacted overnight with a primary anti-His tag antibody (1/1,000; BioLegend), followed by incubation with anti-mouse IgG secondary antibody (1/5,000; Sigma-Aldrich) for 1 h. Immunoblots were visualized using ECL Prime Western Blotting Detection Reagent and an ImageQuant LAS500 system.

### Purification of target proteins

NZ-DnaK, -GroEL, -Pyk, and -GAPDH were cultured in a total of 1 L of GM17cm, and the expression of target proteins was induced as described above. The bacteria were pelleted by centrifugation (3,000 × *g*, 4°C, and 20 min) and then disrupted using a CRYO-PRESS (MicroTech-Nichion, Funabashi, Chiba, Japan). The disrupted cells were suspended in binding buffer (20 mM Na_3_PO_4_∙12 H_2_O, 500 mM NaCl, and 20 mM imidazole in the supernatant) and filtered (0.45 µm). Protein purification was carried out on an AKTA pure protein purification system (GE Healthcare) as previously described (25). Briefly, the cleared cell lysates were loaded onto His-Trap HP 1 mL columns with binding buffer, and the columns were washed with binding buffer (20 column volumes). The resulting samples (supernatant, flow-through, wash, and eluate: F-1 to F-4) were subjected to SDS-PAGE and Western blotting. Fractions containing target proteins were dialyzed against PBS. The concentration of the eluted fractions was determined using a His-tag enzyme-linked immunosorbent assay (ELISA) detection kit (Funakoshi, Tokyo, Japan).

### Tumor necrosis factor (TNF)-α assay in RAW 264.7 cells

RAW 264.7 macrophages were seeded in a 96-well plate at 2 × 10^5^ cells/100 µL/well and cultured at 37°C for 24 h in a 5% CO_2_ environment. RAW 264.7 macrophages were incubated with 100 µL/well of BLF_pH7.3_, ProK-treated BLF_pH7.3_, BLF_pH4.2_, and each of the purified proteins (DnaK, GroEL, Pyk, and GAPDH) for either 2 or 24 h. Subsequently, intracellular levels of transcripts encoding TNF-α and secreted levels of the corresponding proteins were measured using real-time quantitative PCR (RT-qPCR) and ELISA, respectively. RT-qPCR analyses were performed as previously described (27). After 2 h of incubation, total RNA was extracted from RAW 264.7 macrophages using a NucleoSpin RNA Kit (Macherey-Nagel, Düren, Germany) according to the manufacturer’s instructions. Primers for mouse *Actb* and *Tnfa* transcripts (encoding β-actin and TNF-α, respectively) were purchased from TaKaRa Bio, Inc. (Shiga, Japan). Levels of *Actb* were used to normalize transcript concentrations. After 24 h of incubation, the spent medium was collected and assayed for the TNF-α concentration. Commercially available ELISA kits (Thermo Fisher Scientific) were used according to the manufacturer’s instructions.

### Extracellular vesicle isolation and analysis

WT and K56N cultures were incubated at 37°C for 24 h. The bacteria were removed by centrifugation (8,000 × *g*, 4°C, 5 min), and the supernatants were collected. This process was repeated until no pellets were visible, and the resulting supernatants were passed through filters (0.45 µm). The supernatants of WT and K56N were ultracentrifuged (110,000 × *g*, 4°C, 90 min) and collected as EV-free fractions, whereas the pellets containing EVs were suspended in 1.5 mL of PBS. The EV fractions were further centrifuged (3,000 × *g*, 4°C, 15 min) to remove debris, dissolved in 1 mL of PBS, and purified by density gradient centrifugation using layered sucrose solutions (40%, 30%, 20%, 10%) with ultracentrifugation at 110,000 × *g* for 16 h at 4°C. Fractions were collected from the gradient and analyzed using dynamic light scattering to determine particle size and concentration and identify the peak fraction with the highest EV content. The peak fraction was concentrated by ultracentrifugation, resuspended in 100 µL of PBS, filtered through a 0.45 µm spin filter, and analyzed for particle size and concentration using a qNano system (IZON, Addington, Christchurch, NZ). The resulting suspensions were subjected to SDS-PAGE followed by Coomassie brilliant blue (CBB) staining. Bands of interest were excised and submitted for peptide mass fingerprinting analysis (COSMO BIO Co., Ltd., Tokyo, Japan) by MALDI-TOF MS to identify the corresponding proteins. To characterize the shapes of the purified EVs, negative-staining electron microscopy was conducted as previously described (28). EVs suspended in PBS were loaded onto glow-discharged formvar/carbon-coated copper grids and negatively stained with 1% aqueous uranyl acetate. The grids were air-dried and observed using a JEM-1400 transmission electron microscope (JEOL, Tokyo, Japan) operated at 100 kV.

### Anti-inflammatory assay of extracellular vesicles

The anti-inflammatory activity of EVs was assessed using the method of Yamamoto et al. (27). RAW 264.7 cells were pretreated with WT-EVs or K56N-EVs for 24 h and then exposed to 10 ng/mL lipopolysaccharide (LPS) for 24 h. The concentration of TNF-α in the culture supernatants was determined using ELISA kits (Thermo Fisher Scientific), as per the manufacturer’s guidelines.

### Whole-genome sequencing (WGS)

WGS and analysis of the K56N strain were outsourced to Macrogen, Inc. (Seoul, South Korea). Genomic DNA was extracted from K56N strain cells using a DNeasy UltraClean/NoviPure Microbial Kit (Qiagen, Hilden, Germany) according to the manufacturer’s instructions. Sequencing reads were mapped against the publicly available reference genome of the *Lacticaseibacillus rhamnosus* ATCC 53103 strain for variant calling and comparative analysis.

### Statistical analysis

All statistical analyses were performed using Statcel, v.4 (OMS Publishing Inc., Saitama, Japan). *P*-values were calculated using the Tukey-Kramer test to detect statistically significant differences. *P* < 0.05 was considered to indicate a significant difference. Results are presented as the mean and standard deviation (SD).

## RESULTS

### Isolation of LGG-MTs

A total of 197 colonies retained the ability to grow on MRS agar containing streptomycin (256 µg/mL). Satellite colonies and streptomycin-requiring strains were excluded, and the remaining 136 isolates were obtained as MTs (Table 1). The *rpsL* gene encoding ribosomal protein S12 was sequenced to identify 12 types of *rpsL* mutations. The 136 MTs, grouped according to the 12 types of nucleotide mutations, were found to encode mutant S12 proteins with nine distinct amino acid substitutions. Notably, one of the MTs (MT_R99G_) harbored a novel amino acid substitution corresponding to an Arg-to-Gly substitution at residue 99, which was not observed in our previous study (23). To assess streptomycin sensitivity, MT_R99G_ was cultured at 37°C for 16 h in MRS broth containing streptomycin at 64, 128, 256, 512, 1,024, 2,048, 4,096, 8,192, 16,384, 32,768, and 65,536 µg/mL. LGG-MT_R99G_ exhibited a streptomycin MIC of 512 µg/mL.

### Morphological observations

**Fig 1:**
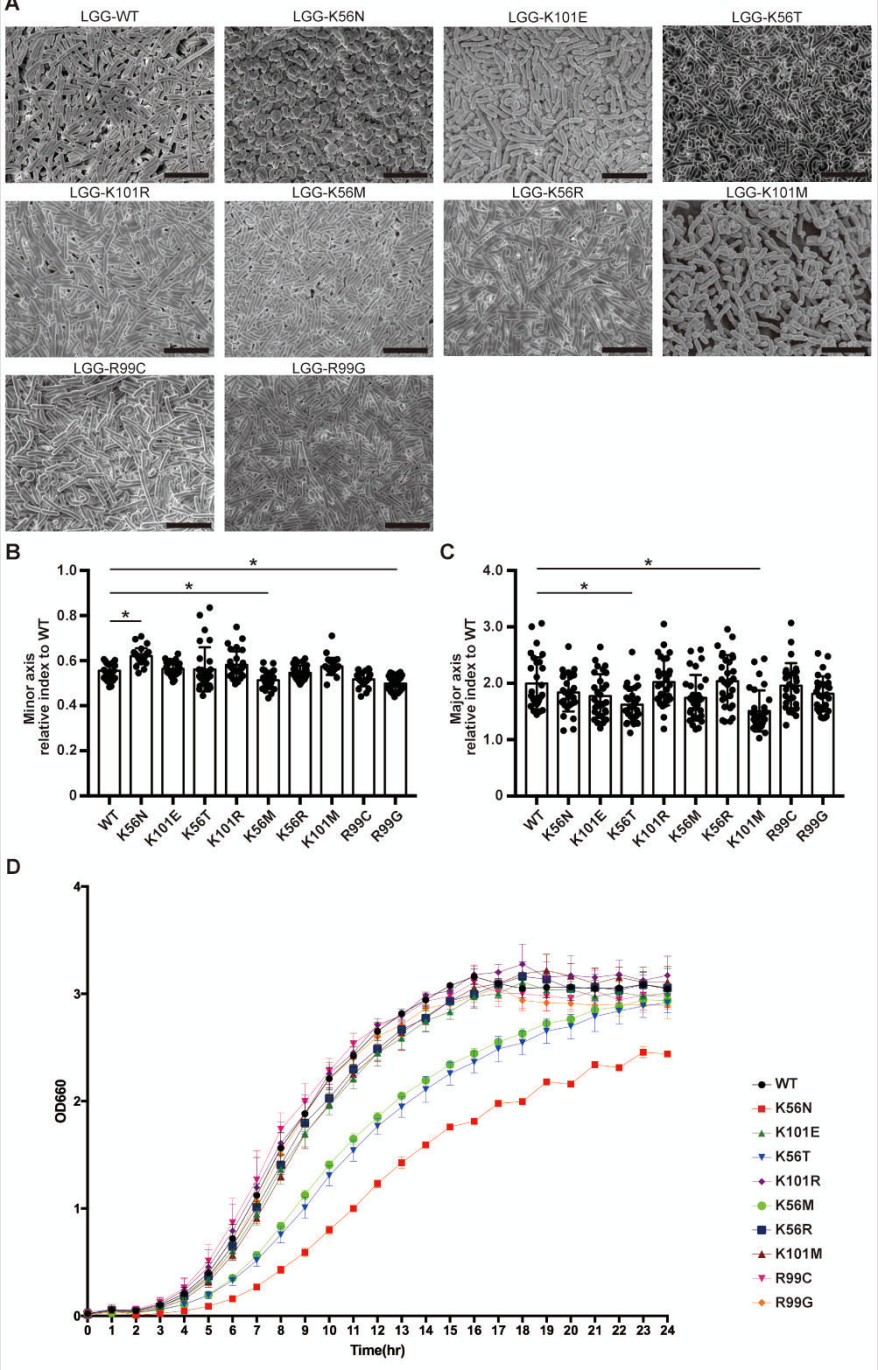
(**A**) Representative SEM images (5,000 × magnification) of WT, MT cells. Bars = 5 µm. (**B and C**) Short and long diameters of each bacterial cell. Data are expressed as mean and SD (*n* = 30). (**D**) Growth curves of WT and MT strains. WT and K56N mutant strains were pre-cultured and inoculated into screw-cap tubes containing fresh medium pre-warmed to 37°C. A spectrophotometer was adjusted to an initial OD_660_ of approximately 0.02. OD_660_ was measured every hour from 0 to 24 h. Growth curves represent the mean OD_660_ value from three independent experiments. Statistical analysis for Fig. 2B was performed using Student’s *t*-test. Before the test, homoscedasticity was verified using the F-test, which confirmed that the variance between groups was equal. Different letters indicate significant differences (*P* < 0.05).

WT and MT strains were cultured for 24 h, and bacterial cell morphology was observed using SEM (Fig. 1A). The shape of the WT was of a linear rod, whereas K56N and K56T were curved rods. K56N had a significantly larger mean short diameter, whereas R99C and R99G had significantly smaller short diameters compared with WT (*P* < 0.05, Fig. 1B). There were no significant differences in the average long diameter (Fig. 1C). The most significant effect appeared to be a decreased growth rate in K56N compared to the wild-type and other MT strains (Fig. 1D).

### Analysis of change in surface protein profiles over time

**Fig 2:**
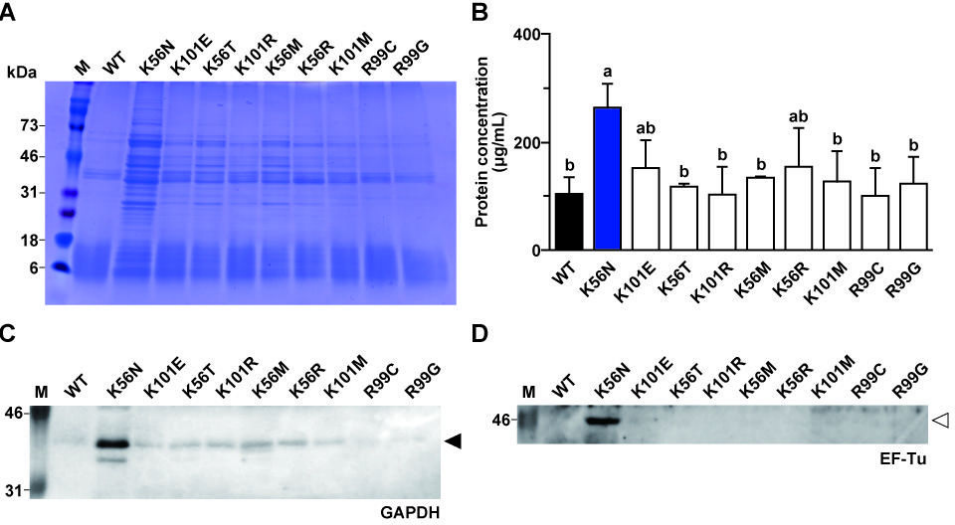
(**A**) Bacterial surface protein profiles of WT and MT strains determined by SDS-PAGE and CBB staining after 24 h incubation (in stationary phase). Equal loading was ensured by normalizing cultures to 1 × 10^9^ CFU/mL prior to BLF preparation. Representative images from three independent experiments are shown. (**B**) Total surface proteins were quantified using the BCA assay. Data are expressed as mean and SD (*n* = 3). Different letters indicate significant differences (*P* < 0.05). (**C and D**) Differential expression of surface GAPDH (**C**) and EF-Tu (**D**) confirmed by Western blotting. Representative images are shown. Black and white arrows indicate GAPDH and EF-Tu, respectively.

WT and MT strains were harvested at 6 h (early log phase), 12 h (late log phase), and 24 h (stationary phase). BLF_pH7.3_ containing bacterial surface proteins was collected, and protein solutions were prepared and subjected to SDS-PAGE with CBB staining to analyze the change in surface protein profiles over time. Few bands were detected in the BLF_pH7.3_ of both the WT and MT strains after 6 h of incubation (Fig. S1). Multiple bands appeared in MT-BLF_pH7.3_ as the incubation time increased (12 h) (Fig. S1). Notably, considerably more bands were observed in K56N-BLF_pH7.3_ than in WT-BLF_pH7.3_ after 24 h of incubation (Fig. 2A).

### Quantification and identification of surface proteins after 24 h incubation

WT and MT strains were cultured for 24 h, after which BLF_pH7.3_ was collected from 1 × 10^9^ CFU of bacteria, and the total protein concentration was determined using the BCA assay. The protein concentration was significantly higher in K56N-BLF_pH7.3_ than in WT-BLF_pH7.3_ (*P* < 0.05, Fig. 2B). WT and MT strains were grown for 24 h, and BLF_pH7.3_ was then collected, lyophilized, and finally subjected to LC-MS analysis. A total of five proteins were detected in WT-BLF_pH7.3_, whereas a total of 23 proteins (the highest number of proteins) were identified in K56N-BLF_pH7.3_ (Table 2). Although GAPDH was consistently detected across all samples by LC-MS, its signal in WT-BLF was below the detection threshold of immunoblotting, reflecting the higher sensitivity of LC-MS compared with Western blotting. The expression of two representative surface proteins, GAPDH and EF-Tu, was analyzed by Western blotting (Fig. 2C and D). Clear bands corresponding to GAPDH (36 kDa) (Fig. 2C) and EF-Tu (43 kDa) (Fig. 2D) were confirmed in K56N.

**TABLE 2:** Surface protein profiles in BLF_pH7.3_^*a*^

Protein name	Molecular mass	WT	K56N	K101E	K56T	K101R	K56M	K56R	R99C	R99G	R99C
Chaperone protein DnaK	67,179		+		+						
Pyruvate kinase	62,920		+		+		+				
60 kDa chaperonin	57,357		+		+						
Uncharacterized protein (A0A5R9D1Q3_LACRH)	51,981					+					
Uncharacterized protein (A0A5R9D1Q3_LACRH)	51,981							+			
Trigger factor	49,875		+		+		+				
30S ribosomal protein S1	47,143				+						
Enolase	47,039		+	+	+		+				
Elongation factor Tu	43,532		+								
Surface antigen (A0A179YJG3_LACRH)	42,714	+	+	+	+	+	+	+	+	+	+
Surface antigen (A0A179YJF0_LACRH)	40,876		+		+						
Glyceraldehyde-3-phosphate dehydrogenase	36,929	+	+	+	+	+	+	+	+	+	
L-lactate dehydrogenase	36,586		+	+	+	+	+	+	+		
Tagatose 1,6-diphosphate aldolase	36,360		+								
ATP-dependent 6-phosphofructokinase	34,176		+								
Class II fructose-1,6-bisphosphate aldolase	31,686				+	+	+		+		
Elongation factor Ts	31,557	+		+	+	+	+	+	+	+	
XRE family transcriptional regulator	31,482				+						
Multidrug ABC transporter ATPase	31,344	+	+					+		+	
Triosephosphate isomerase	27,019		+		+		+				
PTS system beta-glucoside-specific transporter subunit IIABC (Fragment)	26,909					+					
2,3-Bisphosphoglycerate-dependent phosphoglycerate mutase	25,922		+								
Probable DNA-directed RNA polymerase subunit delta	25,541		+								
Ribosome-recycling factor	20,566		+								
L-lactate dehydrogenase	16,706	+								+	
Chromosome segregation protein SMC (Fragment)	16,654							+			
50S ribosomal protein L11	14,871			+			+				
Uncharacterized protein (A0A249N1W2_LACRH)	14,258							+			
Uncharacterized protein (A0A0H0YVH7_LACCA)	14,217							+			
Helix-turn-helix domain-containing protein	14,033		+								
UPF0342 protein BGK71_08085	13,295							+			
50S ribosomal protein L18	13,006				+						
50S ribosomal protein L7/L12	12,582		+	+	+	+		+	+		
30S ribosomal protein S6	11,566		+		+						
10 kDa chaperonin	10,033			+	+			+			
DNA-binding protein HU	9,506		+	+	+		+		+		
Uncharacterized protein (A0A0E3CPJ0_LACRH)	8,551		+		+						
50S ribosomal protein L30	6,788		+	+	+		+	+	+		
Membrane protein	–				+						
Total number of detected protein		5	23	11	22	8	12	13	8	5	1

^*a*^ +, presence of the specific protein; –, absence of the specific protein.

### Transcriptome and metabolome analyses

**Fig 3:**
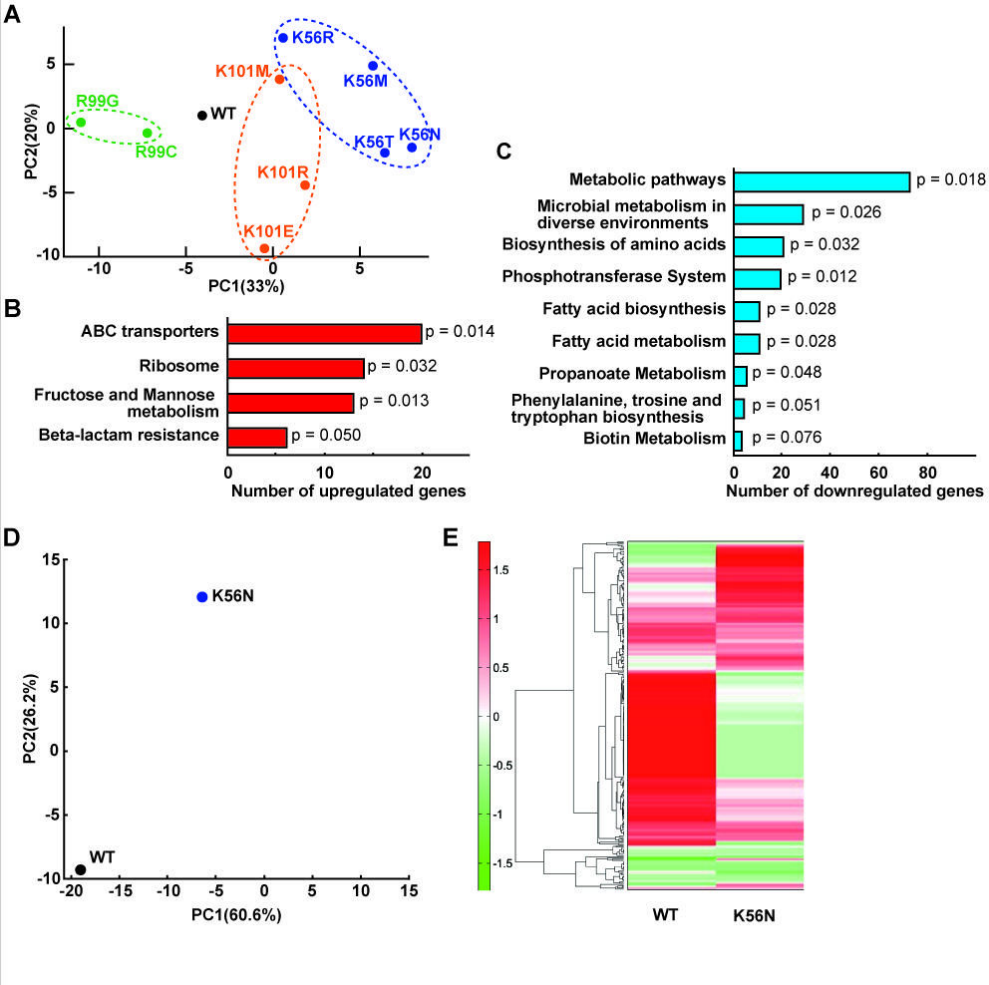
(**A**) PCA score plot of transcriptome profiles of WT and MT strains. PC1 and PC2 indicate the first and second principal component scores, respectively, and numbers in parentheses indicate the percentage contribution of each principal component. (**B and C**) KEGG pathway analysis of upregulated genes (log2 fold change > 1) (**B**) and downregulated genes (log2 fold change < −1) (**C**) in K56N compared with WT. (**D**) PCA plot of metabolome analysis of WT and K56N. (**E**) Heatmap of metabolome analysis of WT and K56N. Vertical axis indicates detected peaks. Distances between peaks are represented by trees. Transcriptome and metabolome analyses were performed once per strain and are presented as exploratory data to illustrate global trends without statistical comparison.

A transcriptome analysis was performed on WT and MT strains after incubation for 24 h. MT strains clustered according to the amino acid mutation sites in the PCA plot (Fig. 3A). KEGG pathway analysis showed that genes associated with ABC transporters, ribosomes, fructose and mannose metabolism, and the beta-lactam resistance pathway were upregulated in K56N (Fig. 3B and C, Table 3). Similarly, WT and MT strains were subjected to metabolome analysis after a 24 h incubation. The PCA plot and heat maps showed significant metabolomic differences between WT and K56N (Fig. 3D and E). Transcriptome and metabolome analyses were performed once for each strain due to resource limitations. These data are therefore presented as exploratory analyses, providing preliminary insights into global transcriptional and metabolic trends rather than definitive statistical comparisons. Table 4 lists the metabolites involved in lactic acid fermentation for which production differed significantly between WT and K56N. Notably, ribulose-5-phosphate, pyruvate, ADT, and ATP were significantly increased (>2-fold) in K56N (Table 4).

**TABLE 3:** DEGs in KEGG pathways in MT_K56N_

Reference pathway	Gene ID	Gene name
Ribosome	LGG_RS04020	16S ribosomal RNA
	LGG_RS11995	23S ribosomal RNA
	LGG_RS04200	30S ribosomal protein S14
	LGG_RS11590	30S ribosomal protein S14
	LGG_RS00075	30S ribosomal protein S18
	LGG_RS07830	30S ribosomal protein S2
	LGG_RS00065	30S ribosomal protein S6
	LGG_RS11705	50S ribosomal protein L13
	LGG_RS08125	50S ribosomal protein L21
	LGG_RS08115	50S ribosomal protein L27
	LGG_RS07990	50S ribosomal protein L28
	LGG_RS09160	50S ribosomal protein L33
	LGG_RS11025	50S ribosomal protein L33
	rplL	50S ribosomal protein L7/L12
ABC transporters	LGG_RS12520	ABC transporter permease
	LGG_RS05735	ABC transporter substrate-binding protein
	LGG_RS01745	Cobalt ABC transporter ATP-binding protein
	LGG_RS02080	Cobalt ABC transporter permease
	LGG_RS09355	Diguanylate cyclase
	LGG_RS09670	Glutamine ABC transporter permease
	LGG_RS13245	Glycerol-3-phosphate ABC transporter permease
	LGG_RS13250	Glycerol-3-phosphate ABC transporter permease
	LGG_RS13240	Glycerol-3-phosphate ABC transporter substrate-binding protein
	LGG_RS10320	Glycine/betaine ABC transporter
	LGG_RS02075	Heme ABC transporter ATP-binding protein
	LGG_RS11585	Manganese transporter
	LGG_RS11580	Membrane protein
	LGG_RS09365	Peptide ABC substrate-binding protein
	LGG_RS09340	Peptide ABC transporter ATP-binding protein
	LGG_RS09345	Peptide ABC transporter ATP-binding protein
	LGG_RS09360	Peptide ABC transporter permease
	LGG_RS05865	Peptide ABC transporter substrate-binding protein
	LGG_RS02290	Phosphate ABC transporter ATP-binding protein
	LGG_RS11660	Sulfonate ABC transporter ATP-binding protein
Fructose and mannose metabolism	LGG_RS06575	6-Phosphofructokinase
	LGG_RS01625	PTS fructose transporter subunit IIA
	LGG_RS04195	PTS glucose transporter subunit IIABC
	LGG_RS13130	PTS mannose transporter subunit IIA
	LGG_RS13125	PTS mannose transporter subunit IIAB
	LGG_RS13135	PTS mannose transporter subunit IID
	srlA	PTS sorbitol transporter subunit IIC
	LGG_RS01995	Hypothetical protein
	LGG_RS01585	Mannose-6-phosphate isomerase
	LGG_RS12850	Rhamnose isomerase
	LGG_RS12860	Rhamnulokinase
	LGG_RS09560	Rhamnulose-1-phosphate aldolase
	LGG_RS13020	Sorbitol-6-phosphate 2-dehydrogenase
Beta-lactam resistance	LGG_RS09355	Diguanylate cyclase
	LGG_RS09365	Peptide ABC substrate-binding protein
	LGG_RS09340	Peptide ABC transporter ATP-binding protein
	LGG_RS09345	Peptide ABC transporter ATP-binding protein
	LGG_RS09360	Peptide ABC transporter permease
	LGG_RS05865	Peptide ABC transporter substrate-binding protein

**TABLE 4:** Metabolites that more than doubled in abundance in K56N

Metabolite	Concentration (pmol/10^9^ cells)		Comparative analysis ratio (K56N vs WT)
	WT	K56N	
Ribulose 5-phosphate	1,425	22,877	16.0
CTP	705	6,584	9.3
CDP	973	6,132	6.3
GDP	793	4,757	6.0
Gln	4,567	25,526	5.6
GTP	2,402	10,659	4.4
Ribose 5-phosphate	369	1,389	3.8
ATP	7,168	25,170	3.5
CMP	3,293	8,987	2.7
Acetyl CoA divalent	645	1,753	2.7
UTP	7,709	20,229	2.6
Inosine	1,559	3,717	2.4
ADP	7,980	17,998	2.3
NADP+	875	1,939	2.2
CoA divalent	316	699	2.2
GMP	5,013	9,882	2.0
Asn	77,062	151,601	2.0

### Adherence to HT-29 cells

**Fig 4:**
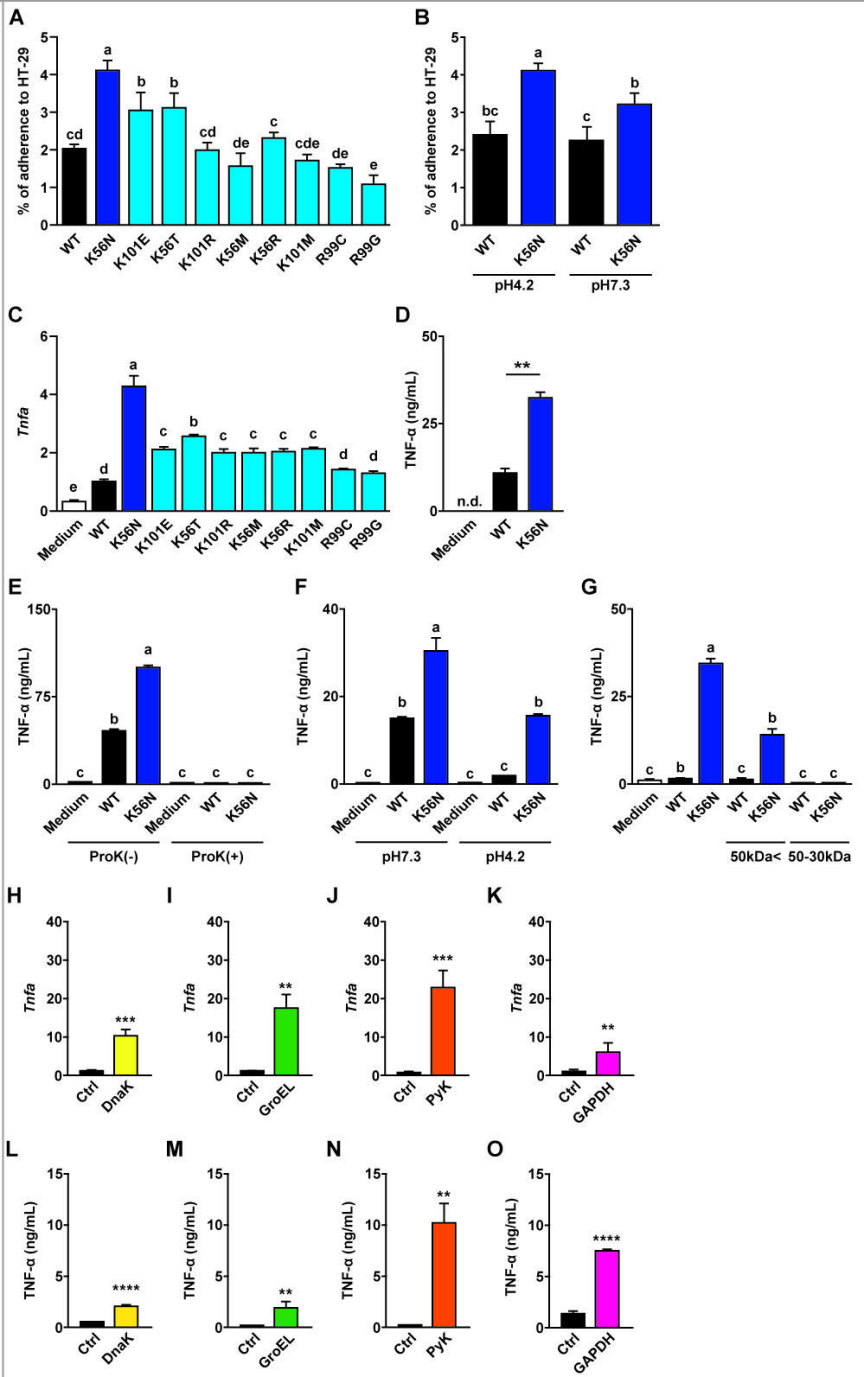
(**A**) Adherence of WT, MT-Cell_pH4.2_ and (**B**) WT, K56N-Cell_pH7.3_ evaluated using HT-29 cells. (**C**) RAW 264.7 cells were treated with WT, MT-BLF_pH7.3_, and TNF-α production was evaluated by RT-qPCR. (**D–G**) RAW 264.7 cells were treated with (**D**) WT, K56N-BLF_pH7.3_, (**E**) ProK-treated WT, K56N-BLF_pH7.3_, (**F**) WT, K56N-BLF_pH4.2_, (**G**) fractionated K56N-BLF_pH7.3_ >50 kDa, and K56N-BLF_pH7.3_ 50 kDa–30 kDa; TNF-α production was evaluated by ELISA. (**H–O**) RAW 264.7 cells were treated with rDnaK, rGroEL, rPyk, and rGAPDH. TNF-α production in stimulated versus nontreated cells was evaluated by RT-qPCR (**H–K**) and ELISA (**L–O**). Representative images from three independent experiments are shown. Data are expressed as mean and SD. Different letters indicate significant differences (*P* < 0.05). ****P < 0.0001; ***P < 0.001; **P < 0.01 vs WT.

Bacterial adherence mediated by surface proteins was examined using HT-29 human colonic epithelial cells. HT-29 cells were incubated with either Cell_pH4.2_ or Cell_pH7.3_, after which the numbers of initial and adherent bacteria were determined by serial dilution, and the adhesion rate was calculated. K56N-Cell_pH4.2_ showed significantly higher adhesion values than WT and all other MTs (*P* < 0.05, Fig. 4A). By contrast, K56N-Cell_pH7.3_ exhibited a significantly lower adhesion rate compared with K56N-Cell_pH4.2_ (*P* < 0.05, Fig. 4B). It should be noted that the specific surface proteins responsible for these adhesion differences were not determined in this study; the results reflect overall phenotypic differences in adherence under the tested conditions.

### Inflammatory effects of surface proteins

Inflammatory effects of surface proteins were investigated using RAW 264.7 mouse macrophages. RAW 264.7 cells were incubated with BLF_pH7.3_, ProK-treated BLF_pH7.3_, or BLF_pH4.2_, and cytokine-encoding gene expression and cytokine secretion were measured using RT-qPCR and ELISA, respectively. Compared with WT-BLF_pH7.3_, *Tnfa* expression was significantly upregulated after incubation of RAW 264.7 cells with K56N-BLF_pH7.3_ (*P* < 0.05, Fig. 4C). Correspondingly, ELISA of the supernatants indicated that TNF-α secretion was increased in K56N-BLF_pH7.3_ (*P* < 0.05, Fig. 4D). No significant increase was detected in RAW 264.7 cells incubated with ProK-treated K56N-BLF_pH7.3_ (Fig. 4E). Incubation of RAW 264.7 cells with K56N-BLF_pH4.2_ resulted in a significant decrease in TNF-α accumulation compared with K56N-BLF_pH7.3_ (*P* < 0.05, Fig. 4F). TNF-α secretion increased significantly when the cells were incubated with K56N-BLF_pH7.3_ >50 kDa, whereas no significant increase was observed in cells incubated with K56N-BLF_pH7.3_ 50–30 kDa (*P* < 0.05, Fig. 4G).

### Identification of immunostimulatory proteins

Effective immunostimulatory molecules were identified using gmLAB. The gene sequence of each potential putative immunomodulatory protein was inserted into plasmid pNZ8148#2 using *Kpn*I, *Bam*HI, and *Hin*dIII digestion to generate the appropriate expression vectors (Fig. S2): pNZ8148#2SEC-DnaK, pNZ8148#2SEC-GroEL, pNZ8148#2SEC-Pyk, and pNZ8148#2CYT-GAPDH. The vectors were then sequenced to confirm the absence of deletions and mutations (Fig. S2). Each expression vector, along with the original control vector, was introduced into strain NZ9000 via electroporation to generate the putative immunomodulatory protein–producing gmLAB (designated NZ-DnaK, NZ-GroEL) along with the control gmLAB (designated NZ-VC). Gene expression was induced in the gmLAB by treatment with nisin, and Western blotting was performed using an anti-His-tag antibody. Bands corresponding to DnaK (73.6 kDa), GroEL (63.2 kDa), Pyk (68.7 kDa), and GAPDH (40.5 kDa) were detected in cellular extracts of nisin-stimulated gmLAB. However, no bands were detected in NZ-DnaK, NZ-GroEL, NZ-PyK, or NZ-GAPDH without nisin stimulation, similar to the control, NZ-VC (Fig. S2). These results indicate that the constructed gmLAB produced the potential putative immunomodulatory proteins following nisin stimulation. The immunostimulatory effects of these proteins were investigated using RAW 264.7 cells incubated with each protein. *Tnfa* expression and TNF-α production were then analyzed using RT-qPCR and ELISA, respectively. Expression of the pro-inflammatory cytokine gene *Tnfa* was significantly increased in RAW 264.7 cells incubated with 100 ng of purified rDnaK, rGroEL, rPyk, or rGAPDH compared with the control (Fig. 4H through K). Moreover, ELISA results indicated that each of the purified proteins promoted the secretion of TNF-α into the culture supernatant (Fig. 4L through O).

### Changes in EVs

**Fig 5:**
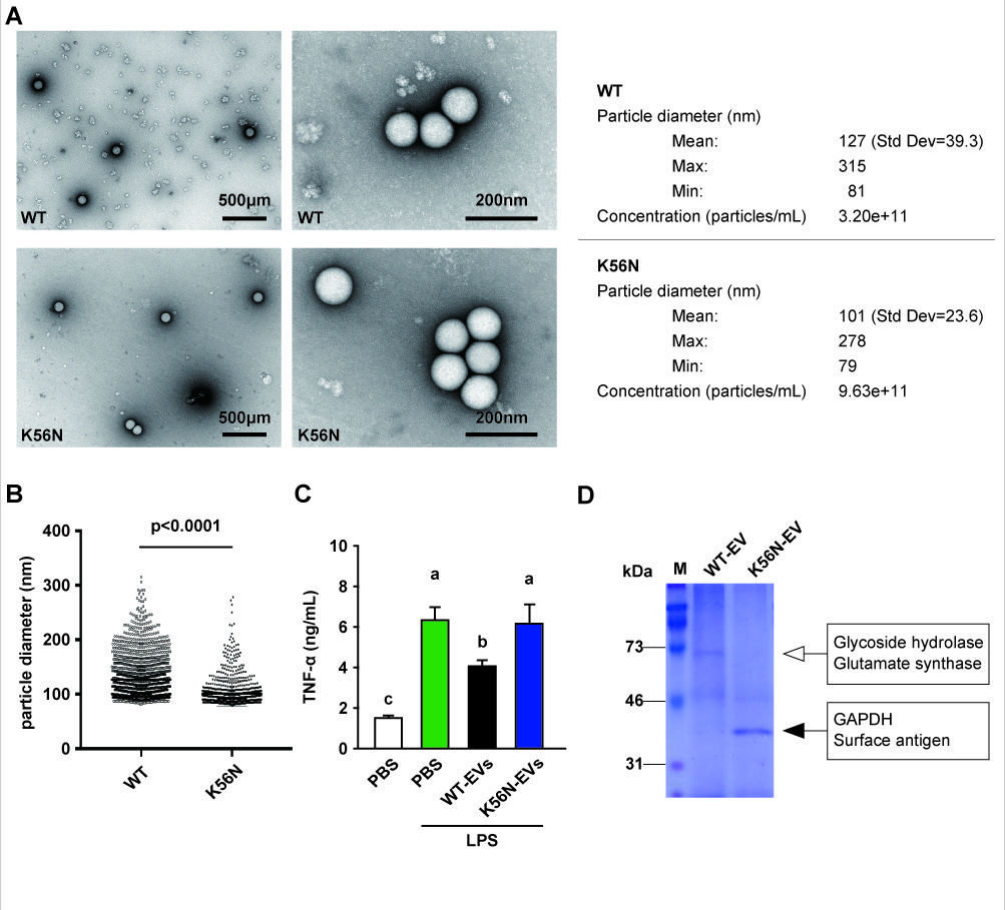
(**A**) Representative TEM images of EVs from WT, K56N. Bars = 500 or 200 nm. The particle diameter of EVs is shown in the table in (**A**) and the dot plot chart in (**B**). (**C**) RAW 264.7 cells were treated with WT-EVs or K56N-EVs for 24 h, followed by stimulation with 10 ng/mL LPS for 24 h before ELISA. (**D**) SDS-PAGE images of WT-EV and K56N-EV, and the results of proteome analysis using LCMS-IT-TOF show the detected protein names. Different letters indicate significant differences (*P* < 0.05). Representative images from three independent experiments are shown. Data are expressed as mean and SD.

The EV fractions were photographed using transmission electron microscopy (TEM), and vesicles that appeared to be EVs were identified in each strain (Fig. 5A). In WT, the measured particle size of the EVs was 100 nm–200 nm (average 161.5 nm) (Fig. 5A and B). K56N exhibited a reduction in EV particle size (average 121.0 nm) and an increase in the number of particles compared with WT (Fig. 5A and B). SDS-PAGE analysis showed bands at approximately 70 kDa in WT-EVs and 31 kDa–46 kDa in K56N-EVs (Fig. 5C). TNF-α secretion was significantly reduced in LPS-stimulated RAW 246.7 cells treated with WT-EVs (Fig. 5D). Conversely, this effect was lost in K56N-EVs (Fig. 5D). The 31 to 46 kDa bands were identified as GAPDH and surface antigens.

## DISCUSSION

RE is a simple, economical, and safe microbial breeding strategy for generating mutants exhibiting enhanced properties. The present work explored the impact of RE through an in-depth analysis of the bacterial proteosurfaceome. Our previous study revealed that K56N exhibited curvilinear growth compared with WT cells (23). Here, we confirmed the presence of a number of curved K56N cells exhibiting a significantly greater short diameter compared with the WT and other MTs, as observed by SEM. While similar morphological changes have been reported in other bacterial species under antibiotic stress (e.g., *Caulobacter crescentus* [29]), the functional implications of the curvature observed in K56N remain to be elucidated.

Notably, while our previous work showed that RE in LGG enhanced mucin adhesion via surface GAPDH expression (23), the present study further reveals that the same K56N mutation alters the composition and immunological function of EVs. Specifically, EVs derived from K56N lose the anti-inflammatory capacity of wild-type EVs, indicating that ribosomal mutations may influence host-microbe interactions through cell-surface changes and EV-mediated mechanisms.

In this study, bacterial surface proteins were extracted by changing the net charge from positive to negative. Several *Lactobacillus crispatus* surface proteins, including GAPDH (pI 5.2), enolase (pI 4.8), glutamine synthetase (pI 5.3), and glucose-6-phosphate isomerase (pI 5.7), are positively charged and electrostatically bound to cell wall components in acidic growth environments, whereas these proteins are negatively charged and released into the medium at alkaline pH (30–32). Similarly, many bacterial moonlighting proteins with pI values ranging from 4.5 to 6.5 (33) are presumed to be associated with the cell wall in a pH-dependent manner. Therefore, we were able to obtain surface moonlighting proteins from BLF_pH7.3_ by washing bacterial cells first with PBS_pH4.2_ (below the pI) and then PBS_pH7.3_ (above the pI) to induce protein dissociation.

Protein profiles in BLF_pH7.3_ were analyzed across different growth phases. SDS-PAGE followed by Coomassie brilliant blue staining revealed that MT strains exhibited a broader diversity of surface-associated proteins over time than the WT strain. Notably, the K56N mutant showed a marked accumulation of surface proteins at 24 h. While these enrichment patterns were reproducible across biological replicates, we acknowledge that the extraction method recovers only a subset of surface-associated proteins and cannot fully exclude the possibility of leakage or structural aberrations. Future studies using intracellular/surface marker proteins and complementary extraction approaches will be required to address this limitation. Previous studies have demonstrated that ribosomal stabilization enhances protein synthesis during the late growth phase in *Streptomyces coelicolor* and *Escherichia coli* strains harboring mutations in *rpsL* (17, 34). The mutant ribosomes in K56N may similarly exhibit increased stability or altered translational efficiency, potentially contributing to enhanced expression or export of surface proteins. Further investigation into the functional consequences of the K56N ribosomal mutation is thus warranted. Although the *rpsL* K56N mutation is likely the primary driver of the observed phenotype, WGS identified additional mutations in the K56N strain. Although none of these mutations were located in genes previously associated with virulence or adhesion, their potential involvement in modulating immune activation or surface protein expression cannot be entirely excluded. Therefore, future studies employing clean, site-directed mutants will be necessary to definitively attribute the observed phenotype to the *rpsL* mutation.

We primarily focused our investigation on the 24 h time point. LC-MS analysis of the WT revealed two *Lactobacillaceae* surface moonlighting proteins: surface antigen (34) and GAPDH (35, 36), in addition to three surface proteins with unknown function: elongation factor thermos stable (37, 38), multidrug ABC transporter ATPase (39), and L-lactate dehydrogenase (38). In K56N, by contrast, we identified more surface moonlighting proteins, including chaperonin protein DnaK (40), pyruvate kinase (41), 60 kDa chaperonin (GroEL) (42), trigger factor (43), enolase (44), EF-Tu (34, 36, 45), and triosephosphate isomerase (36), as well as additional surface proteins with unknown function, including L-lactate dehydrogenase (46) and 50S ribosomal protein L7/L12 (37). These results indicate that the K56N mutation is accompanied by differential protein display on the bacterial cell surface.

We also examined changes in the transcriptome in MT strains using RNA sequencing. MT strains showed unique transcriptome patterns based on the sites of amino acid mutations in ribosomal protein S12. Intriguingly, strains harboring certain mutations at *rpsL* codon 99 (R99C or R99G) exhibited similar proteosurfaceome profiles and a significantly greater short diameter compared with the WT. The correlation between the genotype and corresponding phenotype should be explored in future research. It should be noted that transcriptome and metabolome analyses were performed once for each strain and are therefore considered exploratory, providing preliminary insights rather than definitive statistical conclusions. Notably, in K56N, the expression of ABC transporter pathway proteins was enriched according to KEGG pathway analysis. In a previous study, we consistently found that the level of surface GAPDH in K56N was decreased by inhibition of ABC transporters (23). In addition, we observed that ribosome-related genes were upregulated. In *Streptomyces*, the *rpsL* mutation leads to increased expression of the ribosome recycling factor gene and further enhanced protein synthesis during the late growth phase (47). Taken together, these data suggest that increased surface protein display in K56N could be due to increased translational efficiency and/or activation of protein transport via ABC transporters.

Adherence is critical for the effectiveness of probiotic strains, as it promotes both the competitive exclusion of pathogenic bacteria and the exertion of immunomodulatory effects (48, 49). Some proteins found in MT-BLF_pH7.3_ reportedly function as adhesins when exposed on the cell surface, including GAPDH and EF-Tu, which are involved intracellularly in glycolysis and translation, respectively, as well as DnaK, pyruvate kinase, GroEL, trigger factor, enolase, and triosephosphate isomerase (34–36, 40–45). Indeed, our previous study showed that K56N exhibited cell surface GAPDH accumulation and enhanced adhesion to human colonic mucin (23). Thus, we hypothesized that *rpsL* mutations would enhance bacterial adherence to intestinal cells. When cells were washed with PBS_pH4.2_, which promotes the retention of surface proteins, K56N-Cell_pH4.2_ exhibited increased ability to adhere to HT-29 cells. By contrast, adherence of K56N decreased following treatment with PBS_pH7.3_, promoting surface protein dissociation (K56N-Cell_pH7.3_). These results suggest that K56N strongly adheres to intestinal cells via surface adhesins.

*Lactiplantibacillus plantarum* enolase and *Lactobacillus johnsonii* EF-Tu reportedly modulate cytokine profiles (21, 46). TNF-α is a pro-inflammatory cytokine produced by immune cells (e.g., macrophages and dendritic cells) in response to infection or tissue damage as a means of maintaining homeostasis (50). A previous study suggested that probiotic LAB strains increase production of pro-inflammatory IL-6 and facilitate the differentiation of B cells into plasma cells (51). Another study reported that the administration of probiotic strains prevents the development of ileitis *in vivo* via stimulation of epithelial TNF-α production (52). Therefore, we examined the effects of bacterial surface and secreted proteins on TNF-α secretion in RAW 264.7 cells. Compared with WT-BLF_pH7.3_, K56N-BLF_pH7.3_ exhibited enhanced TNF-α production. However, the effect disappeared when K56N-BLF_pH7.3_ was digested with ProK. Exposure to K56N-BLF_pH4.2_ resulted in less cytokine accumulation than exposure to K56N-BLF_pH7.3_. These findings demonstrate that K56N surface proteins exert immunostimulatory effects by modulating cytokine profiles. Furthermore, we found that rDnaK, rGroEL, rPyk, and rGAPDH increased TNF-α secretion in RAW 264.7 cells. Pneumococcal DnaK, EF-Tu, enolase, and GAPDH have been shown to promote the secretion of TNF-α by macrophages (53, 54). Another study reported that TNF-α production was stimulated when macrophages were incubated with leptospiral GroEL (55). Likewise, K56N-DnaK, GroEL, PyK, and GAPDH may stimulate host immune responses. To the best of our knowledge, this is the first report describing the immunomodulating properties of bacterial PyK. Future research should examine the immunomodulatory effects of these molecules in probiotics. While these findings highlight the functional importance of selected moonlighting proteins, it should be noted that the extraction protocol used in this study recovers only a subset of surface-associated proteins. More comprehensive approaches, such as those reported by Savijoki et al. (56), have identified a much broader surfaceome (56). Our focus here was to highlight changes in moonlighting proteins that are particularly relevant to adhesion and immunomodulatory functions. It should also be noted that while the observed immunostimulatory effects of K56N surface and secreted proteins provide valuable preliminary insights, definitive assignment of specific proteins to these phenotypes will require targeted knockout or overexpression studies in future work. Future studies employing complementary extraction methods will be necessary to capture the full spectrum of LGG surface proteins.

Tong et al. reported that WT-EVs exhibit potent anti-inflammatory properties in a dextran sulfate sodium-induced colitis model via suppression of TNF-α (57). In the present study, we found that the K56N mutation in the *rpsL* gene altered the mechanism of EV secretion, as well as the morphology and protein composition of EVs. Notably, proteomic analysis revealed that EVs from the K56N strain were enriched in several proteins, including GAPDH and surface antigens. These changes likely reflect altered protein secretion, possibly via upregulation of ABC transporter activity, and may underlie the differential immune responses elicited by WT- and K56N-derived EVs. Importantly, functional assays using rGAPDH demonstrated its ability to stimulate TNF-α secretion in RAW 264.7 macrophages, supporting the idea that GAPDH is one of the bioactive components contributing to the enhanced pro-inflammatory activity of K56N-EVs.

Although our ProK treatment experiments support the involvement of surface-associated proteins in the observed macrophage-activating effect, we recognize that this method is non-specific and cannot distinguish between surface-exposed and vesicle-encapsulated proteins, nor completely degrade proteins shielded within the lipid bilayer. Therefore, although the loss of activity upon ProK treatment suggests a role for proteinaceous factors, further studies using gene knockouts or protein-specific inhibition strategies will be essential to confirm the contribution of individual proteins such as GAPDH. These findings reinforce the importance of identifying key effector molecules within EVs. Future studies should employ detailed proteomic and lipidomic profiling, combined with functional EV fractionation techniques, to pinpoint the specific components responsible for modulating cytokine responses and delineate their mechanisms of action.

Probiotic LAB have gained considerable scientific and commercial interest in the fields of human health and animal production. LGG is one of the most widely commercialized and extensively studied probiotic strains and exhibits a variety of effects both *in vitro* and *in vivo*. However, even such beneficial strains exhibit high inter-individual variability, as supported by two studies: a 2001 study by Kalliomäki et al. reporting the effects of LGG on the prevention of atopic dermatitis in children at high risk (58); and a 2006 study by Fölster-Holst et al., which found no significant effects in infants with atopic dermatitis. As ribosome-engineered LGG-MTs can be obtained easily and safely and exhibit enhanced robustness and functionality, they are promising probiotic candidates. Further research is recommended to evaluate their safety and efficacy both *in vitro* and *in vivo*.

In conclusion, this study revealed that the K56N mutation in the *rpsL* gene induces enhanced protein display on the bacterial surface and EV release. The surface proteins mediate stronger bacterial adhesion to intestinal cells and exert immunostimulatory effects against macrophages by modulating cytokine secretion. These findings highlight the potential use of K56N as a probiotic strain with enhanced properties and demonstrate the potential and practicality of RE for developing probiotic strains for industrial use.

## Data Availability

The genome sequencing data generated in this study have been deposited in the DDBJ database under the BioProject accession number PRJDB37402.

## SUPPLEMENTAL MATERIAL

Supplemental material spectrum.02635-25-s0001.pdf Figures S1 and S2; Table S1.

ASM does not own the copyrights to Supplemental Material that may be linked to, or accessed through, an article. The authors have granted ASM a non-exclusive, world-wide license to publish the Supplemental Material files. Please contact the corresponding author directly for reuse.
