# Supplementary material for: Ribosome-engineered Lacticaseibacillus rhamnosus GG with enhanced adhesion and immune activation via surface moonlighting proteins
Source: Microbiol Spectr. 2025 Oct 27;13(12):e02635-25. doi: 10.1128/spectrum.02635-25 (PMC12671100; doi:10.1128/spectrum.02635-25)
Supplement: Supplemental material — Figures S1 and S2; Table S1. [file spectrum.02635-25-s0001.pdf]

Supplemental Materials

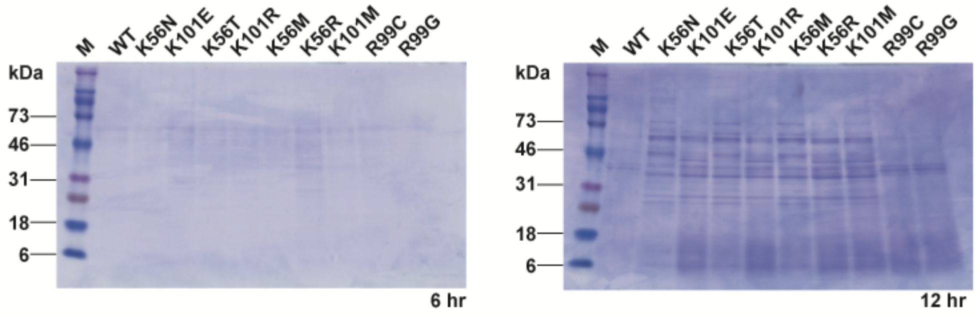

FIG S1. Temporal changes in bacterial surface protein profiles analyzed by SDS-PAGE with CBB staining after 6 h (early log phase) and 12 h (late log phase) of incubation. Representative images from three independent experiments are shown. Equal loading was ensured by normalizing cultures to 1 × 10^9 CFU/mL prior to BLF preparation.

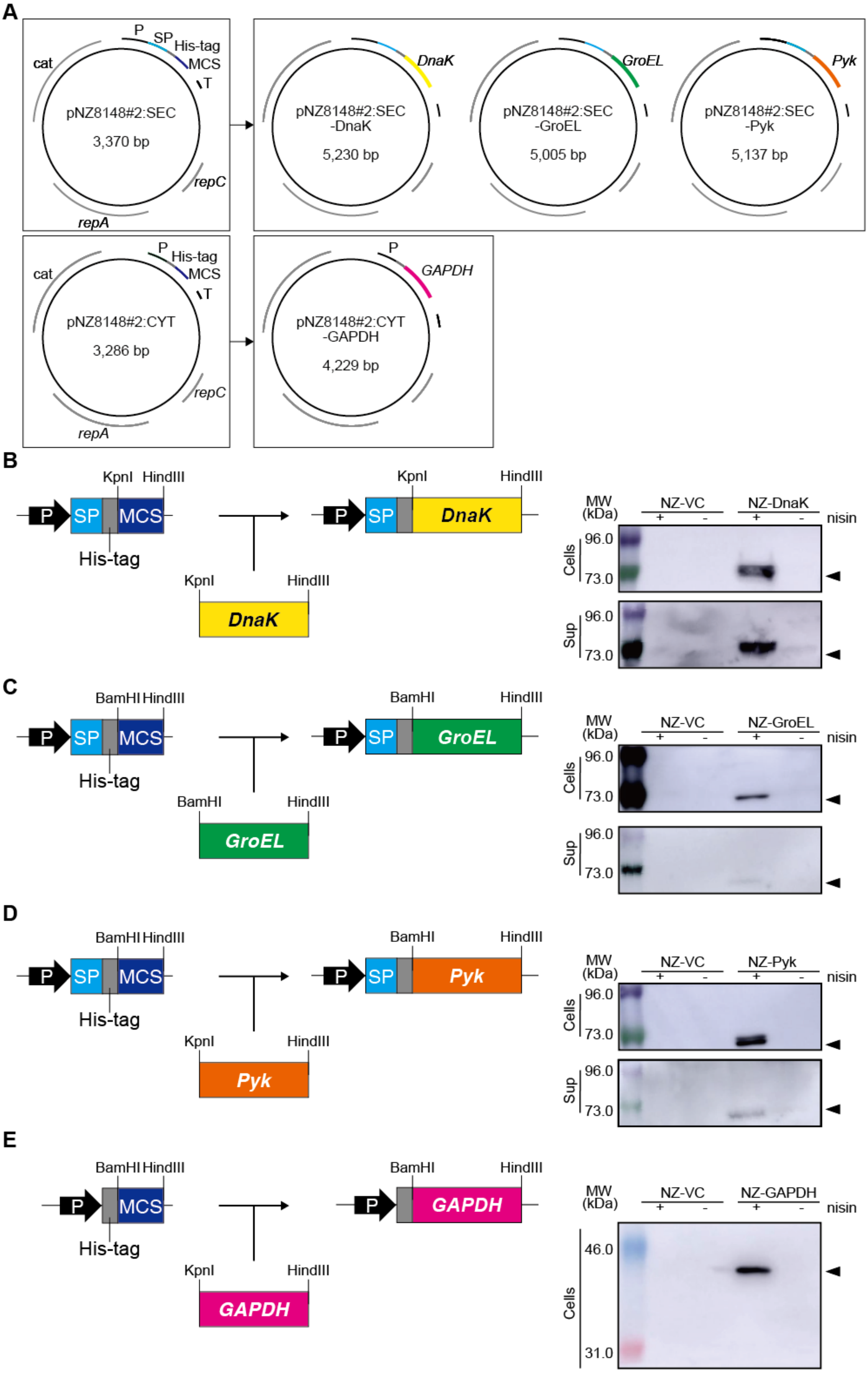

A
P
SP
His-tag
MCS
T
cat
pNZ8148#2:SEC
3,370 bp
repC
repA
DnaK
pNZ8148#2:SEC
-DnaK
5,230 bp
GroEL
pNZ8148#2:SEC
-GroEL
5,005 bp
Pyk
pNZ8148#2:SEC
-Pyk
5,137 bp
pNZ8148#2:CYT
3,286 bp
GAPDH
pNZ8148#2:CYT
-GAPDH
4,229 bp
B
KpnI HindIII
His-tag
DnaK
MW (kDa)
NZ-VC
NZ-DnaK
nisin
+ - + -
Cells
Sup
96.0
73.0
C
BamHI HindIII
GroEL
NZ-GroEL
D
Pyk
NZ-Pyk
E
GAPDH
NZ-GAPDH
46.0
31.0

FIG S2 (A) Nucleotide sequence of each target protein digested with *Bam*HI, *Kpn*I, and *Hin*dIII and inserted into the MSC of the lactococcal expression vector pNZ8148#2 to construct the expression vector, pNZ8148#2SEC and pNZ8148#2CYT. P, nisin-inducible promoter; His-tag, histidine tag; MCS, multi-cloning site; T, terminator; repC and repA, replication origins; cm, chloramphenicol acetyltransferase. DnaK (B), GroEL (C), Pyk (D), and GAPDH (E) gene expression by gmLAB was analyzed by Western blotting using an anti–His-tag antibody. NZ-VC and each protein were cultured with/without nisin, an inducer of gene expression, and cell extracts were analyzed by Western blotting. Arrows indicate bands corresponding to DnaK (73.6 kDa), GroEL (63.2 kDa), Pyk (68.7 kDa), and GAPDH (40.5 kDa), respectively. M, molecular mass marker (kDa); −/+, absence/presence of nisin stimulation.

Table S1. List of all variants identified in the K56N strain compared with the wild-type (WT) by whole-genome sequencing.

Genomic Position	Ref	Alt	Gene Name	Annotation	Mutation
263302	A	G	LRHM_RS01260	missense_variant	T420A
294329	A	G	-	upstream_gene_variant	
302599	T	C	-	upstream_gene_variant	
354397	G	C	LRHM_RS01660	missense_variant	G437A
448995	C	A	-	upstream_gene_variant	
448997	G	Long insertion(#1)	-	upstream_gene_variant	
530251	C	T	LRHM_RS02485	synonymous_variant	F68F
653998	ACGC	A	glpO	disruptive_inframe_deletion	G164deletion
873965	TGCC	T	LRHM_RS04170	conservative_inframe_deletion	G186deletion
1116956	C	T	-	upstream_gene_variant	
1122890	C	A	LRHM_RS05380	missense_variant	D454E
1576468	G	C	LRHM_RS07485	missense_variant	A241G
1688278	A	G	rsgA	synonymous_variant	Y136Y
1989124	A	T	LRHM_RS09380	missense_variant	V903D
2056155	C	T	LRHM_RS09685	stop_gained	W355*
2154813	C	Long insertion(#2)	pglX	disruptive_inframe_insertion	S591_D592insertionKISSGKISFRHVPVGSLF
2157663	A	T	pglX	synonymous_variant	S757S

2157665	A	T	pglX	missense_variant	S757T
2157668	T	TTTGTGG	pglX	conservative_inframe_insertion	R755_N756insertionPQ
2157672	AATAACT	A	pglX	disruptive_inframe_deletion	V753_I754deletion
2157681	A	T	pglX	synonymous_variant	R751R
2157699	A	ATCTTTT	pglX	disruptive_inframe_insertion	F745deletion_insertionLKD
2547013	C	A	rpsL	missense_variant	K56N

Ref: reference allele; Alt: alternative allele in K56N.

#1:GGTGAAAAACTATAAAAGTACTTTAATAATAGGATTTACCAATAGTTCGGCGTATAATAAGTGCAACC

#2:long insertion: CAAAAATATCTAGCGGAAAGATATCCTTTCGACATGTTCCTGTCGGCTCATTGTT
